# Antimicrobial Activity of Nano-GeO_2_/CTAB Complex Against Fungi and Bacteria Isolated from Paper

**DOI:** 10.3390/ijms252413541

**Published:** 2024-12-18

**Authors:** Xu Geng, Yan Wei, Yuanxin Li, Siqi Zhao, Zhengqiang Li, Heng Li, Chen Li

**Affiliations:** 1 Key Laboratory for Molecular Enzymology and Engineering of the Ministry of Education, College of Life Sciences, Jilin University, Changchun 130012, China; gengxu21@mails.jlu.edu.cn (X.G.); weiyan22@mails.jlu.edu.cn (Y.W.); yuanxin22@mails.jlu.edu.cn (Y.L.); lzq@jlu.edu.cn (Z.L.); 2 State Key Laboratory for Diagnosis and Treatment of Severe Zoonotic Infectious Diseases, Key Laboratory for Zoonosis Research of the Ministry of Education, Institute of Zoonosis, College of Veterinary Medicine, Jilin University, Changchun 130062, China; zhaosq23@mails.jlu.edu.cn; 3 Information Center, Jilin Agricultural University, Changchun 130118, China; hengl@jlau.edu.cn

**Keywords:** nano-GeO_2_/CTAB complex, paper protection, antibacterial, antifungal, spore germination

## Abstract

Microbial attack, particularly fungal degradation of cellulose, is a leading cause of paper damage. To address fungal spores and the rising concern of microbial drug resistance, a nano-Germanium dioxide (GeO_2_)/cetyltrimethylammonium bromide (CTAB) complex (nano-GeO_2_/CTAB complex) with potent antibacterial properties was synthesized. Its inhibitory effects were evaluated against bacteria, including Gram-positive *Staphylococcus aureus* and Gram-negative *Escherichia coli*, as well as fungi isolated from paper (*Fusarium* spp., *Aspergillus* spp., and *Penicillium citrinum*). The nano-GeO_2_/CTAB complex exhibited significant (*p* < 0.05) inhibitory effects against *S. aureus* and *E. coli*. Moreover, a 60 min treatment with 1 mg/mL of the complex significantly inhibited the growth of all tested fungi and reduced their biomass after five days of culture, while 4 mg/mL completely deactivated spores. Filter paper pre-treated with the nano-GeO_2_/CTAB complex showed complete resistance to microbial attack, exhibiting no fungal growth and a clear inhibition zone devoid of bacterial growth. In contrast, untreated controls displayed fungal coverage exceeding 95% within five days. These findings highlight the nano-GeO_2_/CTAB complex as a promising antimicrobial agent for protecting paper materials from microbial degradation.

## 1. Introduction

The degradation of paper documents and records poses a significant challenge to preservation efforts [1]. Among the various causes, microbial damage is particularly impactful, as it targets cellulose and hemicellulose, the primary structural components of paper [2,3]. Fungal degradation is noteworthy [4], as fungi thrive in low-nutrient environments and secrete highly active enzymes [5,6]. Common fungi isolated from paper include species of *Penicillium*, *Aspergillus*, *Fusarium*, and *Trichoderma* [7]. These fungi produce cellulose-degrading enzymes (cellulases), which play a central role in breaking down paper cellulose [8], and amylases, xyloglucanases, and gelatinases that further contribute to the degradation process [9,10]. As fungi colonize paper, their extracellular enzymes and hyphae weaken its structure, increasing its fragility [11]. Furthermore, acidic byproducts of fungal metabolism accumulate on paper surfaces, accelerating the cleavage of cellulose chains and compounding the damage [12]. Bacteria such as *Proteobacteria* and *Actinobacteria* can also degrade the cellulose in paper through the cellulase secreted by themselves [1,13]. Moreover, the pathogenic bacteria on the paper surface can even endanger human health and trigger public health issues [14,15].

Methods to combat microbial infestations of paper include physical, chemical, and biological approaches [16]. Physical methods, such as gamma-ray irradiation at doses exceeding 10–20 kGy [17] and freeze-drying, are widely used. However, while high-dose gamma-ray irradiation effectively eliminates microorganisms, it also depolymerizes cellulose chains and accelerates paper aging, limiting its applicability [18,19]. Freeze-drying, though less damaging, is only partially effective against fungal spores [20]. Chemical methods, including liquid fungicides and gas fumigation, are effective but may leave harmful residues or pose risks to human health [21,22]. For example, ethylene oxide (EtO) fumigation can treat moldy paper on a large scale but is highly toxic and prohibited in some countries [23]. Biological methods, such as applying antibiotics to paper surfaces, can eliminate colonizing microorganisms but risk promoting microbial resistance [24,25], while their oxidation products may accelerate paper degradation [26]. Consequently, there is a critical need to develop long-lasting antimicrobial treatments with minimal human health risks.

Recent advances in nano-metal oxide research have broadened their applications in biomedicine and biotechnology [27]. Nano-metal oxides are valued for their antimicrobial and ultraviolet (UV)-protective properties [28,29,30]; however, their strong cohesive forces often result in aggregation in solution, reducing their effectiveness [4]. To address this issue, improving their dispersibility and chemical stability is critical [31]. Surface modification with surfactants such as cetyltrimethylammonium bromide (CTAB) during synthesis has been shown to improve the growth and stability of nano-metal oxide crystals in solution [32,33]. For instance, nano-metal oxides like ZnO [34] and TiO_2_ [35] with high dispersibility and antimicrobial activity have been synthesized using surfactants, while GeO_2_ has demonstrated antibacterial activity against Gram-positive and Gram-negative bacteria [36], although its potential for inhibiting fungi remains unexplored.

To combat paper degradation caused by microbial invasion, a nano-GeO_2_/CTAB complex with high dispersibility and antimicrobial activity was synthesized and characterized using energy-dispersive X-ray spectroscopy (EDS), X-ray diffraction (XRD), and zeta potential analysis. Its inhibitory effects were tested against microorganisms, including Gram-positive and Gram-negative bacteria, as well as three types of fungi that were isolated from paper. The results demonstrate that the nano-GeO_2_/CTAB complex exhibits remarkable antimicrobial activity, presenting a promising strategy for preserving paper-based records and other documents.

## 2. Results and Discussion

### 2.1. Morphology and Elemental Distribution of the Nano-GeO_2_/CTAB Complex

**Figure 1:**
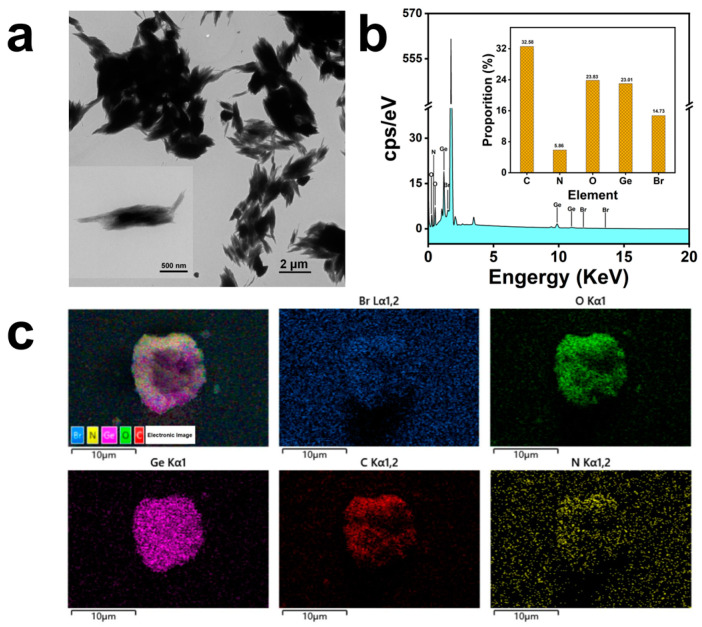
Morphology and element distribution of nano-GeO_2_/CTAB complex; (**a**) TEM morphology of nano-GeO_2_/CTAB complex; (**b**) elements on the surface of nano-GeO_2_/CTAB complex and the content of each element; (**c**) element distribution on the surface of nano-GeO_2_/CTAB complex. Kα and Lα were shown different electron energy levels of the K shell and L shell, respectively.

Figure 1a depicts the morphology of the newly synthesized nano-GeO_2_/CTAB complex at 1000× and 8000× magnifications, respectively. Transmission electron microscopy (TEM) analysis revealed that the nano-GeO_2_/CTAB complex exhibited a spindle-shaped structure composed of nano-GeO_2_/CTAB complex-containing filaments. In contrast, raw GeO_2_ displayed an irregular block-like structure, indicating that the addition of the surfactant CTAB effectively regulated the morphology of the nano-GeO_2_/CTAB complex, confirming its successful synthesis [37].

As shown in Figure 1b, the EDS spectrum highlights the elemental composition of the nano-GeO_2_/CTAB complex, with the *x*-axis representing energy and the *y*-axis representing intensity. The elemental proportions were as follows: carbon (C) 35.52%, nitrogen (N) 5.86%, oxygen (O) 23.83%, germanium (Ge) 23.01%, and bromine (Br) 14.73%. Figure 1c illustrates the distribution of individual elements on the surface of the nano-GeO_2_/CTAB complex, represented as dot maps.

### 2.2. Physicochemical Characterization of the Nano-GeO_2_/CTAB Complex

**Figure 2:**
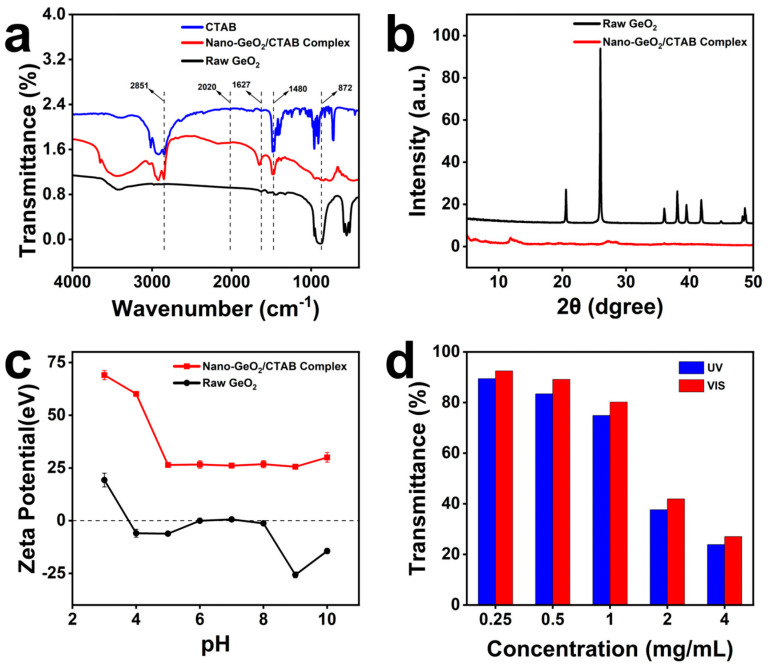
Nano−GeO_2_/CTAB complex characterization; (**a**) nano−GeO_2_/CTAB complex FTIR spectra; (**b**) nano−GeO_2_/CTAB complex XRD characterization; (**c**) nano−GeO_2_/CTAB complex ZETA potential; (**d**) nano−GeO_2_/CTAB complex transmittance.

Fourier transform infrared (FTIR) spectra of raw GeO_2_ and the nano-GeO_2_/CTAB complex were recorded using a Shimadzu 3346 spectrometer for samples prepared using the potassium bromide (KBr) compression method (Figure 2a). The spectra revealed absorption peaks near 2020 cm^−1^ and 872 cm^−1^ for both raw GeO_2_ and the nano-GeO_2_/CTAB complex, attributed to the asymmetric stretching vibration of Ge-O-Ge [38]. Peaks near 1627 cm^−1^, observed in both CTAB and the nano-GeO_2_/CTAB complex, correspond to -NH vibrations [39]. Additionally, absorption peaks near 2851 cm^−1^ and 1627 cm^−1^ were detected in CTAB and the nano-GeO_2_/CTAB complex but were absent in raw GeO_2_, confirming the successful modification of nano-GeO_2_ by CTAB. A peak near 1480 cm^−1^ in both CTAB and the nano-GeO_2_/CTAB complex likely originates from the δas + N−CH_3_ bands of the CTAB headgroup [40].

XRD analysis was conducted using an X-ray diffractometer (Almelo, The Netherlands) with a scanning range of 2*θ* = 5°~50° and Cu Kα radiation (λ = 1.5418 Å) (Figure 2b). Raw GeO_2_ displayed sharp diffraction peaks at 20.5°, 26°, 36°, 38°, 39.5°, and 41.8°, corresponding to the (100), (101), (110), (102), (111), and (200) planes, respectively. The notably strong intensity of the peak attributed to the (101) plane and its relatively narrow half-peak width indicated substantial crystallinity of raw GeO_2_ [41]. In contrast, the nano-GeO_2_/CTAB complex exhibited no distinct diffraction peaks, suggesting that the addition of CTAB disrupted the ordered crystal structure during synthesis, aligning with findings by Lin et al., who reported similar results when synthesizing nano-GeO_2_/CTAB complex with surfactants [42].

Given that the zeta potential is an important indicator of the stability of colloidal dispersion stability [43], zeta potential changes in raw GeO_2_ and the nano-GeO_2_/CTAB complex were measured within the pH 2–10 range in Tris-HCl buffer (Figure 2c). Raw GeO_2_ exhibited positive charges at pH < 4 and negative charges at pH > 4, with optimal stability observed at pH 2. In contrast, the nano-GeO_2_/CTAB complex maintained a positive charge across the pH range of 2–10, with zeta potential values exceeding +30 mV at pH < 5 and +25 mV at pH 5–10, confirming that the nano-GeO_2_/CTAB complex synthesized with CTAB exhibits improved dispersibility compared to raw GeO_2_. This enhanced stability prevents precipitation, enabling the uniform distribution of the nano-GeO_2_/CTAB complex within paper cellulose to support and reinforce its structure while protecting it from degradation.

Since photocatalytic degradation of β-1,4 glycosidic bonds can accelerate the deterioration of cellulose in paper, the transmittance of nano-GeO_2_/CTAB complex solutions at different concentrations was evaluated (Figure 2d). The results show that as the concentration of the nano-GeO_2_/CTAB complex increases, its transmittance decreases. Notably, at a concentration of 1 mg/mL, the nano-GeO_2_/CTAB complex exhibited an average shading rate of 25.12% in the UV range (200–400 nm), which increased to 76.13% at 4 mg/mL. This high UV-shading efficiency effectively mitigates the damaging effects of UV light on paper cellulose. In the visible light (VIS) range (390–780 nm), the nano-GeO_2_/CTAB complex demonstrated a lower shading rate, averaging 19.8% at 1 mg/mL and 58.08% at 2 mg/mL. The lower shading rate in the VIS spectrum ensures that its application on paper does not interfere with the visibility of text or artwork, preserving the paper’s aesthetic and functional qualities.

### 2.3. The Inhibitory Effect of the Nano-GeO_2_/CTAB Complex on Gram-Positive and Gram-Negative Bacteria

**Figure 3:**
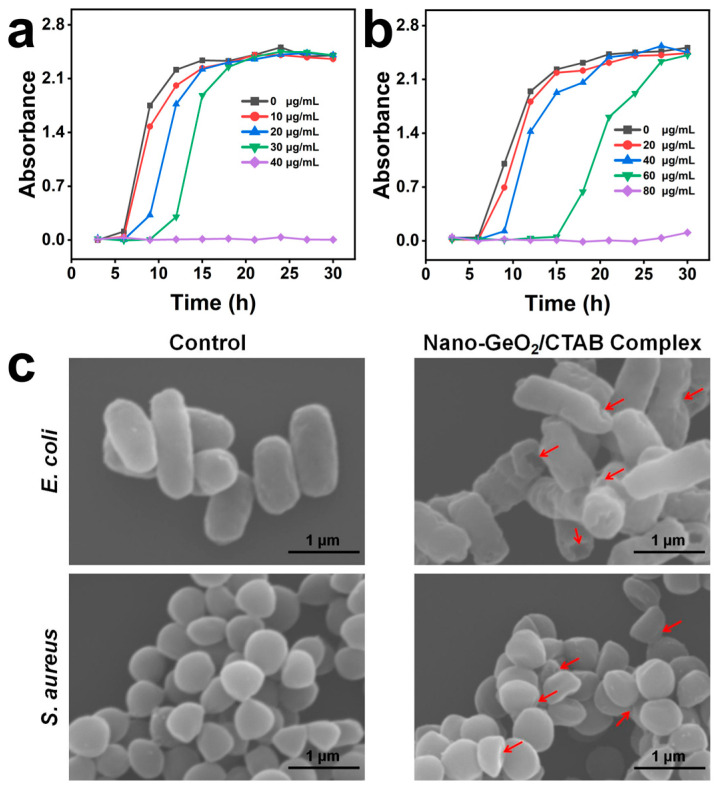
Inhibitory effects of nano−GeO_2_/CTAB complex to Gram−negative and Gram−positive bacteria. (**a**) Growth curve of *E. coli* treated with 0 μg/mL, 10 μg/mL, 20 μg/mL, 30 μg/mL, and 40 μg/mL nano−GeO_2_/CTAB complex for 30 min; (**b**) growth curve of *S. aureus* treated with 0 μg/mL, 20 μg/mL, 40 μg/mL, 60 μg/mL, and 80 μg/mL nano−GeO_2_/CTAB complex for 30 min; (**c**) SEM images of *E. coli* and *S. aureus* treated with 1 mg/mL nano−GeO_2_/CTAB complex at 25 °C for 30 min.

Bacteria in the natural environment can synthesize cellulase to decompose cellulose, contributing significantly to paper degradation [44,45]. In this study, common Gram-positive (*S. aureus*) and Gram-negative (*E. coli*) bacteria were selected to evaluate the antibacterial effects of the nano-GeO_2_/CTAB complex by monitoring their growth curves (Figure 3a,b). Treatment of bacterial cells with varying concentrations of the nano-GeO_2_/CTAB complex (0 μg/mL, 10 μg/mL, 20 μg/mL, 30 μg/mL, and 40 μg/mL) for 30 min resulted in prolonged lag phases and delayed growth for both bacterial species. Notably, as the concentrations of the nano-GeO_2_/CTAB complex increased, *E. coli* cultures required more time to reach an OD_600_ value of 2.0 [46], with a similar trend observed for *S. aureus*.

At nano-GeO_2_/CTAB complex concentrations exceeding 40 μg/mL, *E. coli* was completely inhibited within 30 h, with no detectable regrowth. In contrast, *S. aureus* treated with the same concentration reached an OD_600_ value of approximately 2.0 at 18 h post-treatment, and its growth was not fully inhibited within 30 h at concentrations below 80 μg/mL. These findings indicate that the nano-GeO_2_/CTAB complex exhibits stronger inhibitory effects on *E. coli* compared to *S. aureus*, likely due to structural differences in bacterial cell walls. Gram-negative bacteria (*E. coli*) possess thinner cell walls, making them more susceptible to disruption by the nano-GeO_2_/CTAB complex [47].

SEM analysis further revealed the morphological damage induced by the nano-GeO_2_/CTAB complex on bacterial cell membranes (Figure 3c). Untreated control cells maintained their normal morphology, while cells treated with the nano-GeO_2_/CTAB complex exhibited significant membrane disruption, as indicated by red arrows in Figure 3c. The destructive effects were more pronounced in *E. coli*, which showed extensive membrane collapse, whereas *S. aureus* retained relatively greater cell membrane integrity. These results confirm the enhanced disruptive action of the nano-GeO_2_/CTAB complex against Gram-negative bacteria (*E. coli*), highlighting its potential as an effective antibacterial agent.

### 2.4. Inhibitory Effect of the Nano-GeO_2_/CTAB Complex on Cellulase Activity in Fungi Isolated from Paper

The 16S ribosomal v3-v4 region of bacterial sample IS1894-4 (Figure S1d), along with the internal transcriptional spacer regions of fungal samples IS1894-1 (Figure S1a), IS1894-2 (Figure S1b), and IS1894-3 (Figure S1c), was amplified and sequenced. The sequencing results were identified via BLAST alignment in the NCBI database. The results indicated that the identity between IS1894-4 (Figure S1d) and *Pseudomonas azotoformans* (LT629702.1) was 100%. Moreover, fungal samples IS1894-1, IS1894-2, and IS1894-3 exhibited 100% identity with *Penicillium citrinum* (MH793858.1), *Fusarium verticillioides* (OQ938728.1), and *Aspergillus versicolor* (ON332124.1), respectively.

The antibacterial effects of different concentrations of the nano-GeO_2_/CTAB complex on the three fungal types were investigated by monitoring their growth at OD_600_ after treatment with the nano-GeO_2_/CTAB complex. The results obtained after treatment with concentrations of 0 mg/mL, 0.5 mg/mL, 1 mg/mL, 2 mg/mL, and 4 mg/mL for 60 min followed by culturing for 72 h revealed a progressive delay in germination time for spores from *Fusarium*, *Aspergillus*, and *P. citrinum* as the concentration increased. This delay may be attributed to the induced dormancy of some fungal spores under unfavorable conditions, with gradual germination observed as culture time increased (Figure S2a–c). At a concentration of 4 mg/mL, no fungal spores germinated after 60 min of treatment and 72 h of culturing, indicating that 4 mg/mL is the minimum inhibitory concentration (MIC) for all three fungal species.

Further analysis showed differences in fungal growth dynamics. Maximum OD_600_ values were approximately 3.0 for *Fusarium*, while *Aspergillus* and *P. citrinum* exhibited maximum OD_600_ values between 0.2 and 0.25. These variations likely reflect differences in the time required for each species to produce spores and proliferate in liquid culture. Subsequent experiments were focused on the inhibitory effects of 1 mg/mL of the nano-GeO_2_/CTAB complex on fungal spores.

**Figure 4:**
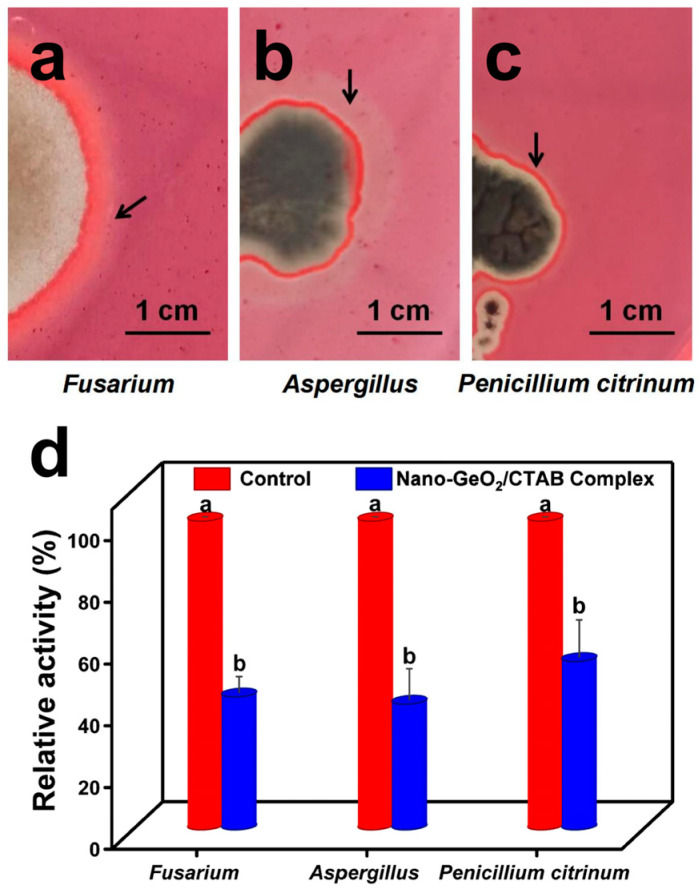
Effects of nano−GeO_2_/CTAB complex treatment on the cellulase activity of *Fusarium*, *Aspergillus*, and *P. citrinum*. (**a**) Cellulase decomposition area of *Fusarium*; (**b**) cellulase decomposition area of *Aspergillus*; (**c**) cellulase decomposition area of *P. citrinum*; (**d**) effects of nano−GeO_2_/CTAB complex treatment on the cellulase activity of *Fusarium*, *Aspergillus,* and *P. citrinum*. When the same bacterial strain is treated with nano−GeO_2_/CTAB complex, different letters on each bar indicate a significant difference in cellulase activity (*p* < 0.05).

Fungi rely on the secretion of cellulase to degrade paper cellulose. To confirm cellulase secretion by *Fusarium*, *Aspergillus*, and *P. citrinum*, cellulose medium was used. The results demonstrated clear zones around colonies on cellulose medium (as shown in arrow), confirming their ability to secrete cellulase and degrade cellulose (Figure 4a–c). Figure 4a–c and Figure S3a confirm that *Pseudomonas*, as a type of bacterium, is also capable of secreting cellulase in vitro and decomposing and utilizing cellulose.

Additionally, the effect of a 60 min treatment with the nano-GeO_2_/CTAB complex on cellulase activity was studied (Figure S4). The results showed a significant reduction in cellulase activity for all three fungal species following treatment with the complex (*p* < 0.01) (Figure 4d). This indicates that the nano-GeO_2_/CTAB complex effectively reduces fungal cell viability and cellulase secretion, mitigating the destructive effects of cellulase on paper cellulose. Figure S3 demonstrates that the treatment of the nano-GeO_2_/CTAB complex has a significant effect on the cellulase activity of *Pseudomonas*.

### 2.5. Resistance of Nano-GeO_2_/CTAB Complex-Treated Paper to Fungal and Bacterial Infestation

**Figure 5:**
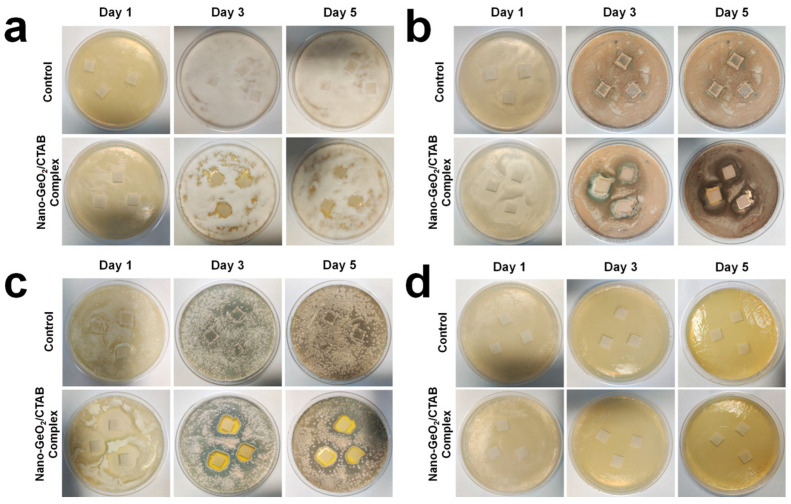
Resistance of filter paper treated with nano−GeO_2_/CTAB complex to the attack of fungi and bacteria. (**a**) Resistance of filter paper treated with nano−GeO_2_/CTAB complex to *Fusarium* attack; (**b**) resistance of filter paper treated with nano−GeO_2_/CTAB complex to *Aspergillus* attack; (**c**) resistance of filter paper treated with nano−GeO_2_/CTAB complex to *P. citrinum* attack. (**d**) Resistance of filter paper treated with nano−GeO_2_/CTAB complex to *Pseudomonas* attack.

Most fungi produce cellulases, enabling them to degrade cellulose and accelerate paper breakdown. To evaluate the resistance of filter paper treated with the nano-GeO_2_/CTAB complex to fungal attack, experiments were conducted using *Fusarium*, *Aspergillus*, and *P. citrinum*. The results showed that after five days of growth, nano-GeO_2_/CTAB complex-treated filter paper exhibited significant resistance to fungal infection compared to the untreated control. For *Fusarium*, untreated filter paper was more than 99% covered by fungal growth by the third day, with no antifungal zone observed (Figure 5a), whereas treated filter paper showed no *Fusarium* infection and exhibited an antifungal zone on the third day. Up to the fifth day, an inhibition zone against Fusarium still existed around the filter paper treated with the nano-GeO_2_/CTAB complex, indicating long-lasting antifungal activity provided by the nano-GeO_2_/CTAB complex.

For *Aspergillus*, untreated filter paper was completely infected by the third day (Figure 5b). By comparison, treated filter paper showed no fungal growth and an antifungal zone on the third day, which remained on the fifth day, demonstrating sustained antifungal efficacy.

When tested against *P. citrinum*, the untreated filter paper was fully colonized by the third day (Figure 5c). In contrast, treated filter paper exhibited an antifungal zone on the third day, which slightly decreased by the fifth day. These results highlight the strong inhibitory effects of the nano-GeO_2_/CTAB complex on all three fungal species, with the greatest resistance observed against *P. citrinum*.

To assess resistance against bacterial infestation, *Pseudomonas*, a Gram-negative bacterium known to produce cellulase [48,49,50] was studied. Untreated filter paper showed no antibacterial zone (Figure 5d), while treated filter paper exhibited an antibacterial zone on the first day. On the fifth day, no *Pseudomonas* infection was observed (Figure 5d). These findings demonstrate that the nano-GeO_2_/CTAB complex effectively inhibits *Pseudomonas* growth and protects the cellulose in paper from bacterial degradation.

### 2.6. Inhibitory Effect of the Nano-GeO_2_/CTAB Complex on Fungal Spores Isolated from Paper

**Figure 6:**
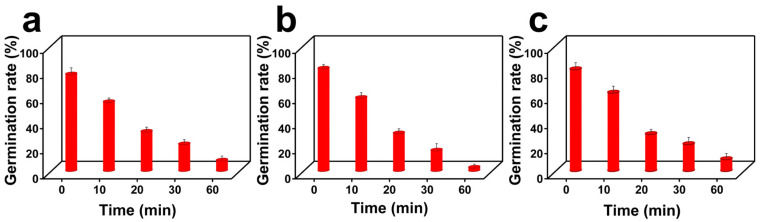
Effects of treatment with nano−GeO_2_/CTAB complex on the spore germination rate of *Fusarium*, *Aspergillus*, and *P. citrinum*. (**a**) Effects of treatment with nano−GeO_2_/CTAB complex for 0 min, 10 min, 20 min, 30 min, and 60 min on the germination rate of *Fusarium* spores; (**b**) effects of treatment with nano−GeO_2_/CTAB complex for 0 min, 10 min, 20 min, 30 min, and 60 min on the germination rate of *Aspergillus* spores; (**c**) effects of treatment with nano−GeO_2_/CTAB complex for 0 min, 10 min, 20 min, 30 min, and 60 min on the germination rate of *P. citrinum* spores.

The effects of different concentrations of the nano-GeO_2_/CTAB complex on the spores of *Fusarium*, *Aspergillus*, and *P. citrinum* were evaluated. The results revealed that increasing treatment time significantly decreased the germination rates of spores from all three fungi. After 60 min of treatment, the germination rates of *Fusarium*, *Aspergillus*, and *P. citrinum* spores were reduced to 8.33%, 2.66%, and 9.33%, respectively, representing statistically significant differences compared to the blank control group (*p* < 0.01) (Figure 6a–c).

### 2.7. Effects of Treatment with Different Concentrations of the Nano-GeO_2_/CTAB Complex on the Biomass of Three Types of Fungi Isolated from Paper

**Figure 7:**
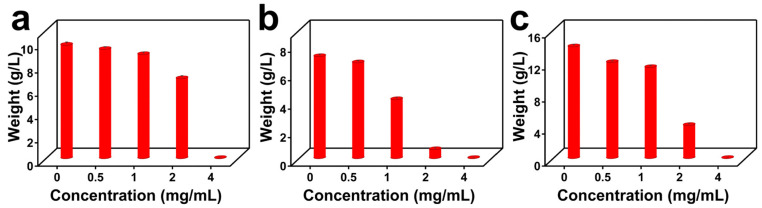
Effects of treatment with different concentrations of nano−GeO_2_/CTAB complex on the biomass of *Fusarium*, *Aspergillus*, and *P. citrinum*. (**a**) Effects of treatment with different concentrations of nano−GeO_2_/CTAB complex on the biomass of *Fusarium*; (**b**) effects of treatment with different concentrations of nano−GeO_2_/CTAB complex on the biomass of *Aspergillus*; (**c**) effects of treatment with different concentrations of nano−GeO_2_/CTAB complex on the biomass of *P. citrinum*.

The effects of different concentrations of the nano-GeO_2_/CTAB complex on the biomass of *Fusarium*, *Aspergillus*, and *P. citrinum* were evaluated. The results showed that fungal biomass decreased as the concentration of the nano-GeO_2_/CTAB complex increased. After treatment with 0 mg/mL, 0.5 mg/mL, 1 mg/mL, 2 mg/mL, and 4 mg/mL for 60 min followed by culturing under suitable conditions for five days, a dose-dependent reduction in biomass was observed for all three fungal types (Figure 7a–c). Notably, at a concentration of 4 mg/mL, no fungal spore germination occurred, and the biomass of *Fusarium*, *Aspergillus*, and *P. citrinum* was reduced to zero, indicating complete bactericidal. Additionally, treatment with 1 mg/mL of the nano-GeO_2_/CTAB complex resulted in a significant reduction in fungal biomass compared to the control group (*p* < 0.01). These findings suggest that the nano-GeO_2_/CTAB complex effectively inhibits fungal growth in a concentration-dependent manner, with 4 mg/mL being sufficient to eliminate *Fusarium*, *Aspergillus*, and *P. citrinum*.

## 3. Materials and Methods

### 3.1. Experimental Materials

*E. coli* (ATCC 25922) and *S. aureus* (ATCC 25923) were obtained from ATCC (Manassas, VA, USA). Glucose was purchased from Amresco (Solon, OH, USA). Zn (NO_3_)_2_, MgSO_4_·7H_2_O, NaOH, and HCl were purchased from Beijing Chemical Works (Beijing, China). Germanium dioxide (raw GeO_2_)_,_ cetyltrimethylammonium bromide (CTAB), sodium chloride, and peptone were obtained from Aladdin (Hangzhou, China). Dipotassium phosphate, dihydrogen potassium phosphate, and disodium hydrogen phosphate were purchased from Sinopharm Chemical Reagent Co., Ltd. (Shanghai, China), and yeast extract was sourced from Sigma-Aldrich (St. Louis, MO, USA). All other reagents were acquired from commercial sources and used without further purification. A Cellulase (CL) Activity Assay Kit was purchased from Beijing Solarbio Science & Technology Co., Ltd. (Beijing, China). All solutions were prepared using water with a resistivity of 18.2 MΩ·cm, generated using a Milli-Q system (Merck Millipore, Waltham, MA, USA).

### 3.2. Synthesis of the Nano-GeO_2_/CTAB Complex

The nano-GeO_2_/CTAB complex was synthesized using a hydrothermal method, following the procedures of Liang Xin and Han Lei [37]. First, raw GeO_2_ (0.9 M) and NaOH solution (0.3 M) were added to 10 mL of ultrapure water and stirred overnight at 25 °C. Subsequently, 12 mL of 0.3 M CTAB solution was poured into the mixture and stirred for 6 h. After stirring, the resulting solution was transferred to a Teflon bottle and heated at 100 °C for 56 h to allow full growth of the GeO_2_ crystals. After the synthesis of the nano-GeO_2_/CTAB complex, it was centrifuged at 8000× *g*, and the precipitate was collected. The separation and purification methods of the nano-GeO_2_/CTAB complex were as follows: Under the condition of room temperature (25 °C), the complex was first centrifuged and washed with 50 mL of ultrapure water at 8000× *g* three times. Subsequently, it was centrifuged and washed with 50 mL of absolute ethanol at 8000× *g* another three times to remove impurities from the product, and then the precipitate was collected. Finally, the obtained precipitate was vacuum-dried at room temperature (25 °C) for 48 h to obtain the nano-GeO_2_/CTAB complex.

### 3.3. Characterization of the Nano-GeO_2_/CTAB Complex

Scanning electron microscopy (SEM) images of the nano-GeO_2_/CTAB complex were obtained using a JSM-6010LA microscope (Japan Electronics Co., Ltd., Tokyo, Japan). XRD analysis was performed using an X-ray diffractometer with a scanning range of 2*θ* = 5°~50° and Cu Kα radiation (λ = 1.5418 Å) (Malvern PANalytical B.V., Almelo, The Netherlands). Infrared (IR) spectra of samples prepared using the potassium bromide (KBr) method were recorded using a SHMADZU 3346 instrument, collecting data in the range of 4000 cm^−1^ to 950 cm^−1^ (Bruker, Bremen, Germany). The transmittance of the nano-GeO_2_/CTAB complex was measured using a SHMADZU UV-2700 spectrophotometer (Shimadzu, Tokyo, Japan). Zeta potential analysis of raw GeO_2_ and nano-GeO_2_/CTAB complex was conducted using an MS 3000 laser particle size analyzer (Malvern Panalytical Ltd., Worcestershire, UK). For EDS, a 2 mg/mL nano-GeO_2_/CTAB complex solution was prepared in ultrapure water, dripped onto a clean silicon wafer, and allowed to dry overnight. After drying was complete, the sample was analyzed using the JSM-6010LA microscope (Japan Electronics Co., Ltd., Tokyo, Japan).

### 3.4. Evaluating the Inhibitory Effects of the Nano-GeO_2_/CTAB Complex on Gram-Negative and Gram-Positive Bacteria

*E. coli* and *S. aureus* cultured at 37 °C with shaking at 180 rpm for 12 h were centrifuged at 3000× *g* for 10 min at 4 °C. The precipitates were then collected and washed thoroughly with sterile PBS. The resulting *E. coli* and *S. aureus* suspensions were diluted to a concentration of 10^6^ CFU/mL. Then, the nano-GeO_2_/CTAB complex was added into 1 mL 10^6^ CFU/mL *E. coli* bacterial solution to achieve 0 μg/mL, 10 μg/mL, 20 μg/mL, 30 μg/mL, and 40 μg/mL, respectively, and treated for 30 min. Similarly, the nano-GeO_2_/CTAB complex was added into 1 mL of the 10⁶ CFU/mL *S. aureus* bacterial suspension to reach final concentrations of 0 μg/mL, 20 μg/mL, 40 μg/mL, 60 μg/mL, and 80 μg/mL, respectively, for antibacterial treatment for 30 min as well. After treatment, the bacterial suspensions were inoculated into 50 mL of Luria broth (LB) at a volume ratio of 1:1000 and incubated at 37 °C with shaking at 180 rpm. At 3 h intervals, 1 mL of bacterial culture was removed, and its optical density at 600 nm (OD_600_) was measured.

### 3.5. Isolation and Identification of Microorganisms on Paper

Following the method of Pavlovic, Jelena et al. [51] for isolating and identifying microorganisms on paper surfaces, the isolation process was as follows: Isolation of microorganisms on the paper surface: Sterilized cotton swabs dipped in sterile ultrapure water were used to gently wipe the surface of the paper with bacterial plaques. Then, the cotton swabs were placed into 2 mL of sterile ultrapure water for dilution. The diluted solution was subjected to the streak plate method on potato dextrose agar (PDA) medium to isolate three kinds of fungi (IS1894-1, IS1894-2, IS1894-3) and one type of bacteria (IS1894-4). Identification of strains: The isolated fungi and bacteria were used for sequencing of the internal transcribed spacer (ITS) and 16S rDNA. Subsequently, the sequencing results were identified via BLAST alignment in the NCBI database.

### 3.6. Evaluating the Effects of Different Concentrations of the Nano-GeO_2_/CTAB Complex on the Growth of Three Types of Fungi Isolated from Paper

To prepare fungal spores, 10 mL of sterile water containing 0.05% Tween-20 was added to solid media cultures of *Fusarium*, *Aspergillus*, and *P. citrinum* grown on PDA for 3 days. The spores were scraped using disposable coating rods, collected, and washed three times with sterile water. The spore suspensions were diluted to a concentration of 10^6^ CFU/mL for each fungal type followed by treatment with the nano-GeO_2_/CTAB complex at concentrations of 0 mg/mL, 0.5 mg/mL, 1 mg/mL, 2 mg/mL, and 4 mg/mL for 60 min. After treatment, the suspensions were inoculated into 50 mL of modified Martin liquid medium at a 1:1000 ratio and incubated at 37 °C with shaking at 180 rpm. Samples (1 mL) were taken every 6 h; then, OD_600_ values of samples were measured.

### 3.7. Evaluating the Inhibitory Effect of Nano-GeO_2_/CTAB Complex on Cellulase Activity of Fungi Isolated from Paper

*Fusarium*, *Aspergillus*, and *P. citrinum* cells were inoculated onto cellulose solid medium and cultured for three days. Once the fungal colonies were fully developed, 0.05% fuchsin solution was added to agar surfaces followed by incubation for 10 min. Following staining, the plates were washed three times with 1 M NaCl to remove excess dye; then, the transparent area surrounding each fungal colony was observed and recorded as an indicator of cellulase activity. Cellulase activity was further quantified using the Cellulase (CL) Activity Assay Kit (Solarbio, Beijing, China), following the manufacturer’s instructions.

### 3.8. Evaluating the Resistance of Filter Paper Treated with the Nano-GeO_2_/CTAB Complex to Fungal and Bacterial Infection

The resistance of filter paper treated with the nano-GeO_2_/CTAB complex to fungal attack was assessed using the following procedure: sterile 1-cm × 1-cm squares of filter paper were soaked in 1 mg/mL nano-GeO_2_/CTAB complex for 20 min and then dried at 40 °C for later use. Three types of fungi (*Fusarium*, *Aspergillus*, and *P. citrinum*) isolated from the paper were cultured on modified Martin agar medium for 3 days and then suspended in sterile PBS using an inoculating loop to generate fungal suspensions. Next, 20 µL of each suspension was spread onto the modified Martin agar medium; then, the treated filter paper was placed onto the surface of the inoculated medium. Plates were inverted and incubated at 30 °C for 5 days; then, the resistance of the treated filter paper to fungal infection was observed and recorded.

### 3.9. Evaluating the Inhibitory Effect of the Nano-GeO_2_/CTAB Complex on Fungal Spores Isolated from Paper at Different Treatment Times

To prepare fungal spores, 10 mL of sterile water containing 0.05% Tween-20 was added to solid PDA media of *Fusarium*, *Aspergillus*, and *P. citrinum* cultured for 3 days. The spores were scraped using disposable coating rods, collected, and washed three times with sterile water; then, spore suspensions for each fungal type were diluted to a concentration of 10^6^ CFU/mL in sterile water. Next, the suspensions were treated with 1 mg/mL nano-GeO_2_/CTAB complex for 0 min, 10 min, 20 min, 30 min, and 60 min. After treatment, the spores were cultured in modified Martin liquid medium for 12 h; then, spore morphology was observed using an optical inverted microscope, and germination was recorded when the length of the spore bud exceeded the spore diameter.

### 3.10. Evaluating the Inhibitory Effect of Different Concentrations of the Nano-GeO_2_/CTAB Complex on Biomass Production by Three Types of Fungi Isolated from Paper

To prepare fungal spores, 10 mL of sterile water containing 0.05% Tween-20 was added to solid PDA cultures of *Fusarium*, *Aspergillus*, and *P. citrinum* followed by incubation for 3 days. The spores were scraped using disposable coating rods, collected, and washed three times with sterile water. Next, the spore suspensions were diluted to a concentration of 10^6^ CFU/mL for each fungal type; then, the diluted suspensions were treated with nano-GeO_2_/CTAB complex at concentrations of 0 mg/mL, 0.5 mg/mL, 1 mg/mL, 2 mg/mL, and 4 mg/mL for 60 min. After treatment, the suspensions were inoculated into 50 mL of modified Martin liquid medium at a 1:1000 ratio and incubated with shaking (180 rpm) at 37 °C for 5 days. At the end of the culture period, the fungal biomass was collected by filtration, dried at 60 °C, and weighed to evaluate the inhibitory effect of the nano-GeO_2_/CTAB complex on biomass production.

### 3.11. Statistical Analysis

All experiments were performed in triplicate, and the results are expressed as mean ± SD. Data analysis and graphing were conducted using GraphPad Prism 10.1.2 (GraphPad Software Inc., San Diego, CA, USA) and Origin 2024 (Origin 2024, OriginLab, Northampton, MA, USA) software. Statistical significance was assessed using analysis of variance (ANOVA) followed by Tukey’s multiple comparisons testing, with significance set at *p* < 0.05. Different letters such as a, b, c, d, and so on marked on the bar charts indicate that there are significant differences between groups after treatment with the nano-GeO_2_/CTAB complex, while the same letters suggest that there are no significant differences between groups [52].

## 4. Conclusions

Achieving effective antifungal treatment, particularly of fungal spores, has long been a challenging task. The novelty of this study lies in the preparation of a nano-GeO_2_/CTAB complex modified by CTAB and possessing high antimicrobial activity; for the first time, nano-GeO_2_/CTAB complex was applied to inhibit three types of fungi (*Fusarium*, *Aspergillus*, and *P. citrinum*) that were isolated from paper. The results demonstrated that the nano-GeO_2_/CTAB complex possesses excellent dispersibility and has a significant damaging effect on the cell membranes of *E. coli* and *S. aureus*. The minimum bactericidal concentrations of the nano-GeO_2_/CTAB complex against *E. coli* and *S. aureus* were 40 μg/mL and 80 μg/mL, respectively. The nano-GeO_2_/CTAB complex significantly reduced cellulase activity in *Fusarium*, *Aspergillus*, and *P. citrinum*, effectively mitigating their ability to degrade cellulose. Notably, treatment with 1 mg/mL of the nano-GeO_2_/CTAB complex for 60 min inhibited spore germination and reduced growth rates and biomass after five days of culture. Complete bactericidal of fungal spores (at 10^6^ CFU/mL) was achieved using a 4 mg/mL concentration of the complex. Filter paper treated with the nano-GeO_2_/CTAB complex exhibited strong resistance to infection by fungi (*Fusarium*, *Aspergillus*, and *P. citrinum*) and bacteria (*Pseudomonas*). After five days, untreated control paper was over 95% covered with fungi, while treated paper showed no fungal or bacterial infection. Antimicrobial zones persisted around the treated paper, further demonstrating the complex’s efficacy in antimicrobial. These findings suggest that the nano-GeO_2_/CTAB complex prepared in this study shows great promise as an effective microbial inhibitor, with significant potential to protect paper and other cellulose-based materials from fungal and bacterial degradation.

## Data Availability

Data are contained within the article or Supplementary Material.

## Supplementary Materials

The following supporting information can be downloaded at: https://www.mdpi.com/article/10.3390/ijms252413541/s1.
