# Supplementary material for: Antimicrobial Activity of Nano-GeO2/CTAB Complex Against Fungi and Bacteria Isolated from Paper
Source: Int J Mol Sci. 2024 Dec 18;25(24):13541. doi: 10.3390/ijms252413541 (PMC11676970; doi:10.3390/ijms252413541)
Supplement: Supplementary file 1 [file ijms-25-13541-s001.zip › ijms-3353965-supplementary.pdf]

Supporting Information

Antimicrobial Activity of Nano-GeO_2/CTAB Complex Against Fungi and Bacteria Isolated from Paper

Xu Geng [1†], Yan Wei [1†], Yuanxin Li [1], Siqi Zhao [2], Zhengqiang Li [1], Heng Li [3], Chen Li [2*]

1 Key Laboratory for Molecular Enzymology and Engineering of the Ministry of Education, College of Life Sciences, Jilin University, Changchun 130012, China; gengxu21@mails.jlu.edu.cn (X.G);weiyan22@mails.jlu.edu.cn (Y.W); yuanxin22@mails.jlu.edu.cn(Y.L); lzq@jlu.edu.cn (Z.L);
2 State Key Laboratory for Diagnosis and Treatment of Severe Zoonotic Infectious Diseases, Key Laboratory for Zoonosis Research of the Ministry of Education, Institute of Zoonosis, and College of Veterinary Medicine, Jilin University, Changchun 130062, China. zhaosq23@mails.jlu.edu.cn (Z.Q);
3 Information center, Jilin Agricultural University, Changchun 130118, P.R. China; hengl@jlau.edu.cn(H.L);
* Correspondence: lc2018@jlu.edu.cn (C.L); Tel: +86-431-8783-6710 (C.L.);
† These authors contributed equally to this work.

Additional Figures: S1-S4

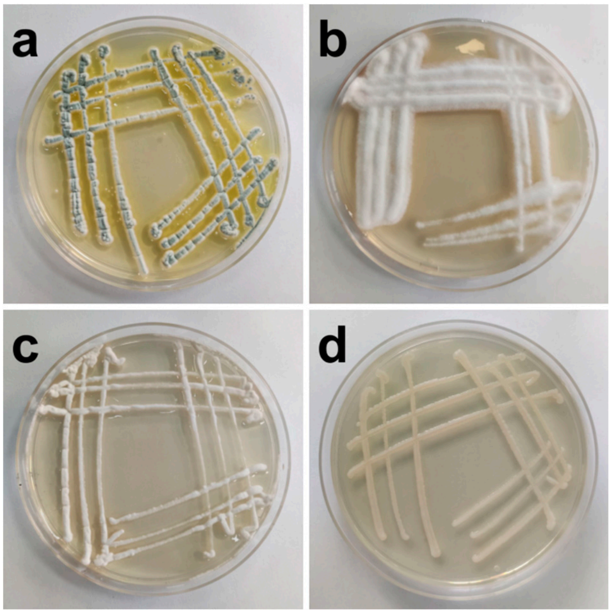

Figure S1. Microorganisms isolated from paper; (**a**) *Penicillium citrinum*; (**b**) *Fusarium*; (**c**) *Aspergillus*; (**d**) *Pseudomonas*.

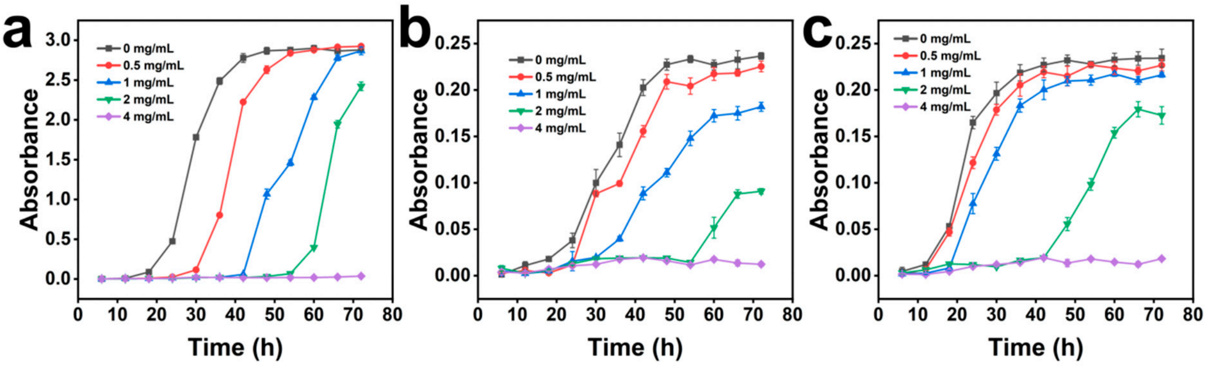

Figure S2. Inhibitory effects of treatment with different concentrations of nano-GeO_2/CTAB complex to *Fusarium*, *Aspergillus* and *Penicillium citrinum*. (**a**) Inhibitory effect of treatment with different concentrations of nano-GeO_2/CTAB complex on *Fusarium*; (**b**) Inhibitory effect of treatment with different concentrations of nano-GeO_2/CTAB complex on *Aspergillus*; (**c**) Inhibitory effect of treatment with different concentrations of nano-GeO_2/CTAB complex on *Penicillium citrinum*.

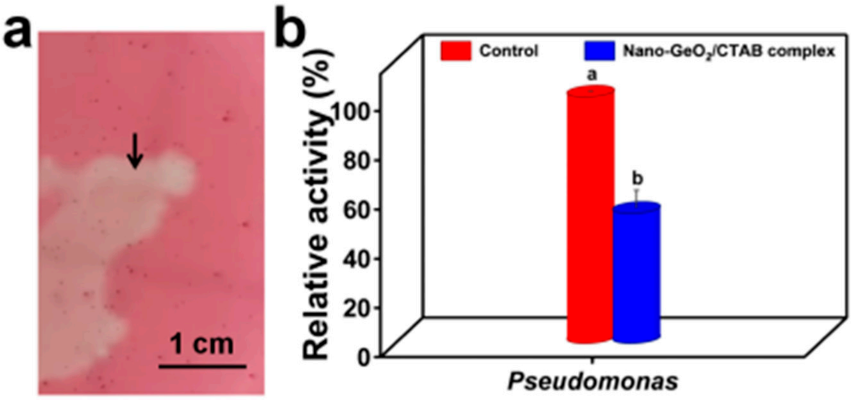

Figure S3. Effects of nano-GeO2/CTAB complex treatment to the cellulase activity of *Pseudomonas*. (**a**) Cellulase decomposition area of *Pseudomonas*; (**b**) Effects of nano-GeO2/CTAB complex treatment on the cellulase activity of *Pseudomonas*.

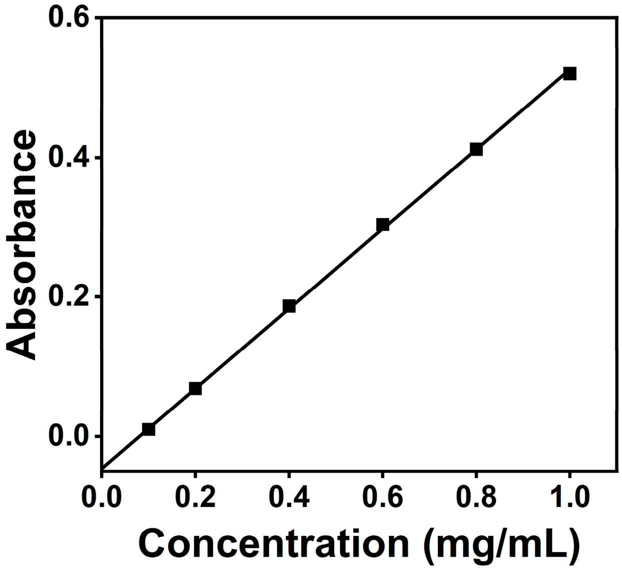

Figure S4. Standard curve of cellulase activity
